# Urinary incontinence rehabilitation of after radical prostatectomy: a systematic review and network meta-analysis

**DOI:** 10.3389/fonc.2023.1307434

**Published:** 2024-03-22

**Authors:** Kai Yu, Fan Bu, Tengteng Jian, Zejun Liu, Rui Hu, Sunmeng Chen, Ji Lu

**Affiliations:** ^1^ Department of Urology, The First Hospital of Jilin University, Changchun, China; ^2^ Department of Plastic and Aesthetic Surgery, The First Affiliated Hospital of Jilin University, Changchun, China

**Keywords:** radical prostatectomy, pelvic floor muscle, urinary incontinence, network meta-analysis, rehabilitation

## Abstract

**Purpose:**

The aim of this study is to provide treatment for patients with urinary incontinence at different periods after radical prostatectomy.

**Methods:**

The PubMed, Embase, Cochrane, and Web of Science were searched for all literature on the effectiveness on urinary control after radical prostate cancer between the date of database creation and 15 November 2023 and performed a quality assessment. A network meta-analysis was performed using RevMan 5.3 and Stata 17.0 software and evaluated using the surface under the cumulative ranking curve.

**Results:**

The results of the network meta-analysis showed that pelvic floor muscle therapy including biofeedback with professional therapist–guided treatment demonstrated better results at 1 month to 6 months; electrical stimulation, biofeedback, and professional therapist guidance may be more effective at 3 months of treatment; professional therapist–guided recovery may be less effective at 6 months of treatment; and combined therapy demonstrated better results at 1 year of treatment. During the course of treatment, biofeedback with professional therapist–guided treatment may have significant therapeutic effects in the short term after surgery, but, in the long term, the combination of multiple treatments (pelvic floor muscle training+ routine care + biofeedback + professional therapist–guided treatment + electrical nerve stimulation therapy) may address cases of urinary incontinence that remain unrecovered long after surgery.

**Conclusion:**

In general, all treatment methods improve the different stages of functional recovery of the pelvic floor muscles. However, in the long term, there are no significant differences between the treatments. Given the cost-effectiveness, pelvic floor muscle training + routine care + biofeedback + professional therapist–guided treatment + electrical nerve stimulation therapy within 3 months and pelvic floor muscle + routine care after 3 months may be a more economical option to treat urinary incontinence.

**Systematic review registration:**

https://www.crd.york.ac.uk/prospero/display_record.php?RecordID=331797, identifier CRD42022331797.

## 1 Introduction

Prostate cancer, one of the most serious diseases affecting older men, has been increasing in incidence year by year in recent years and is now the most common malignancy of the male urological system and related malignancies (1). Prostate cancer may show great heterogeneity among different patients. Active detection is usually adopted for some low- to medium-risk tumors with slow growth, weak invasiveness, and localized prostate cancer. For these tumors that will not develop in a long period of time, radical surgery may bring about great side effects (2). For advanced prostate cancer that progresses rapidly and is highly aggressive, radical prostate cancer surgery is usually used. The most used surgical procedures for radical prostate cancer include standard open retropubic radical prostatectomy, therapeutic laparoscopic radical prostatectomy, and robot-assisted radical prostatectomy. In a radical prostatectomy, a patient’s pelvic floor muscles and the nerves that innervate them may be destroyed, resulting in certain complications, the most common of which is urinary incontinence in patients after radical prostatectomy (3). In the realm of surgical approaches, robot-assisted radical prostatectomy has demonstrated superior outcomes in postoperative urinary control. The research by Sehgal et al. indicates that robot-assisted radical prostatectomy exhibits better results in urinary continence 3 months postoperatively compared with open radical prostatectomy (4). In the assessment of postoperative urinary continence, robot-assisted radical prostatectomy surpasses the outcomes of laparoscopic radical prostatectomy. Regarding the surgical approach, the study by Tuğcu et al. (5) suggests that the perineal approach for radical prostatectomy yields superior results in terms of urinary continence compared with the abdominal approach.

Urinary incontinence often has a negative impact on the patient’s physical and mental health, increasing the patient’s psychological burden and prolonging the postoperative recovery time. Thus, finding more effective and convenient methods is the primary issue in this area. Modern studies have documented that pelvic floor muscle training after radical prostate cancer surgery can improve incontinence, but they have been based on conventional randomized controlled trials and traditional meta-analyses, with no direct-evidence–based medical evidence for the effectiveness of combining many modalities in the treatment of incontinence. In summary, this study used a network meta-analysis to compare the efficacy of many incontinence prevention measures on urinary incontinence. This method allows for a simultaneous comparison of the clinical efficacy of many prevention measures on urinary incontinence prevention, ranked according to the different treatment effects, and thus provides good-evidence–based medical evidence for clinical urologists in preventing urinary incontinence after radical prostate cancer surgery.

## 2 Methods

### 2.1 Study design

The study protocol was registered in the International Prospective Register of Systematic Reviews database (PROSPERO: CRD42022331797). This study followed the updated Preferred Reporting Items for Systematic Reviews and Meta-analyses reporting guideline and its extension for network meta-analysis (6).

### 2.2 Literature search

**Figure 1:**
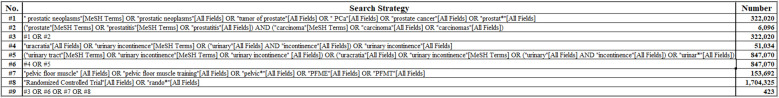
PubMed search strategy.

We searched PubMed, Embase, and Cochrane CENTRAL databases to identify reports published by 15 November 2023, on training for recovery from urinary incontinence after radical prostate cancer surgery. The trial included treatment related to pelvic floor muscle therapy after radical prostate cancer surgery. A number of subject terms and free words related to prostate cancer, radical surgery, multiple pelvic floor muscle training, and randomized controlled trials were used. A detailed database search strategy is given in **Figure 1**.

### 2.3 Inclusion and exclusion criteria

A trial was included in the systematic review if: ① The study type is randomized controlled trial (RCT). ② Languages are limited to English. ③ Disease diagnostic criteria are authoritative and have been published in the literature in professional journals. ④ The data presented in the literature are more standard data. The interventions were pelvic floor training with a physiotherapist or routine care and more. ⑤ The outcome indicator was the number of people recovering from incontinence at 1 month, 3 months, 6 months, and 12 months after pelvic floor training. ⑥ Patients were excluded if they had medical history of urethral, vesical, or prostatic surgery; overactive bladder; and neurogenic lower urinary tract dysfunction. ⑦ Incontinence was controlled by the following criteria: 24-h urine pad <2 g or 5.5 g and 8 g; use 1 or fewer pee pads per day and ICIQ-SF score of 0.

A trial was exclusion in the systematic review if: ① The literature has a high degree of similarity or is a duplicate report. ② The study design in the literature has more obvious flaws. ③ The data included in the literature is incomplete or too much is missing. ④ Animal studies and research. ⑤ Data in the literature were displayed in icon format and data could not be extracted after attempts or data were not available.

Preliminary screening initial screening of ineligible reports was performed on the basis of the title and abstract of the article. Potentially relevant reports were reviewed in the full text of the article, and their relevance was confirmed after data extraction. The screening of titles and abstracts and the screening of full text were done independently by two investigators (ZL and TJ), disagreements were resolved by consensus among the co-authors (KY), and consensus was reached among the authors.

### 2.4 Data collection

Two researchers (FB and RH) independently gleaned the following details from the included articles: the primary author’s name, year of publication, surgical approach, age of the experimental and control groups, criteria for assessing urinary incontinence resolution in each article, total postoperative treatment duration, specific treatment methods and their durations, sample size of the experimental and control groups, as well as the number of patients who achieved urinary continence recovery at 1 month, 3 months, 6 months, and 12 months. Any disparities in data extraction were resolved through consensus among all authors.

### 2.5 Quality evaluation

After undergoing systematic training, the two researchers (FB and RH) independently carried out literature screening and cross-referenced the data extraction in accordance with pre-defined inclusion and exclusion criteria and data extraction forms. If an agreement could not be reached, then a third researcher involved in the study was consulted for mediation. The assessment of bias risk and the quality of the literature was conducted using RevMan 5.3, utilizing the risk of bias assessment criteria outlined in the Cochrane Collaboration Network. The assessment involved evaluating whether: 1) random allocation methods were employed; 2) there was concealment of the allocation scheme; 3) patients and physicians involved were blinded; 4) researchers recording the results were blinded; 5) outcome data were complete; 6) study results were selectively reported; and 7) there were other sources of bias. All literature was independently evaluated by two researchers, and any discrepancies were either further discussed or resolved through consultation with a third researcher co-investigating the study. Funnel plots were employed to ascertain the presence of a small sample effect, with statistical significance set at p < 0.05.

### 2.6 Statistical analysis

Stata 17.0 software was applied for data analysis in this paper. Odds ratios (ORs) and 95% confidence intervals (CI) were used as effect size indicators for the dichotomous outcome indicators. The results of direct comparisons were compared with the results of indirect comparisons using the nodal analysis model in the software to see if the results were consistent. Inconsistency tests were performed on the closed loop formed by the direct and indirect evidence to produce an inconsistency factor (IF). The surface area under the cumulative ranking curve (SUCRA) was used to estimate the probability of treatment for each outcome (7), using the SUCRA to reflect the ranking of the intervention; the closer to 100%, the higher the probability that the intervention is most effective.

## 3 Results

### 3.1 Literature screening process and results

**Figure 2:**
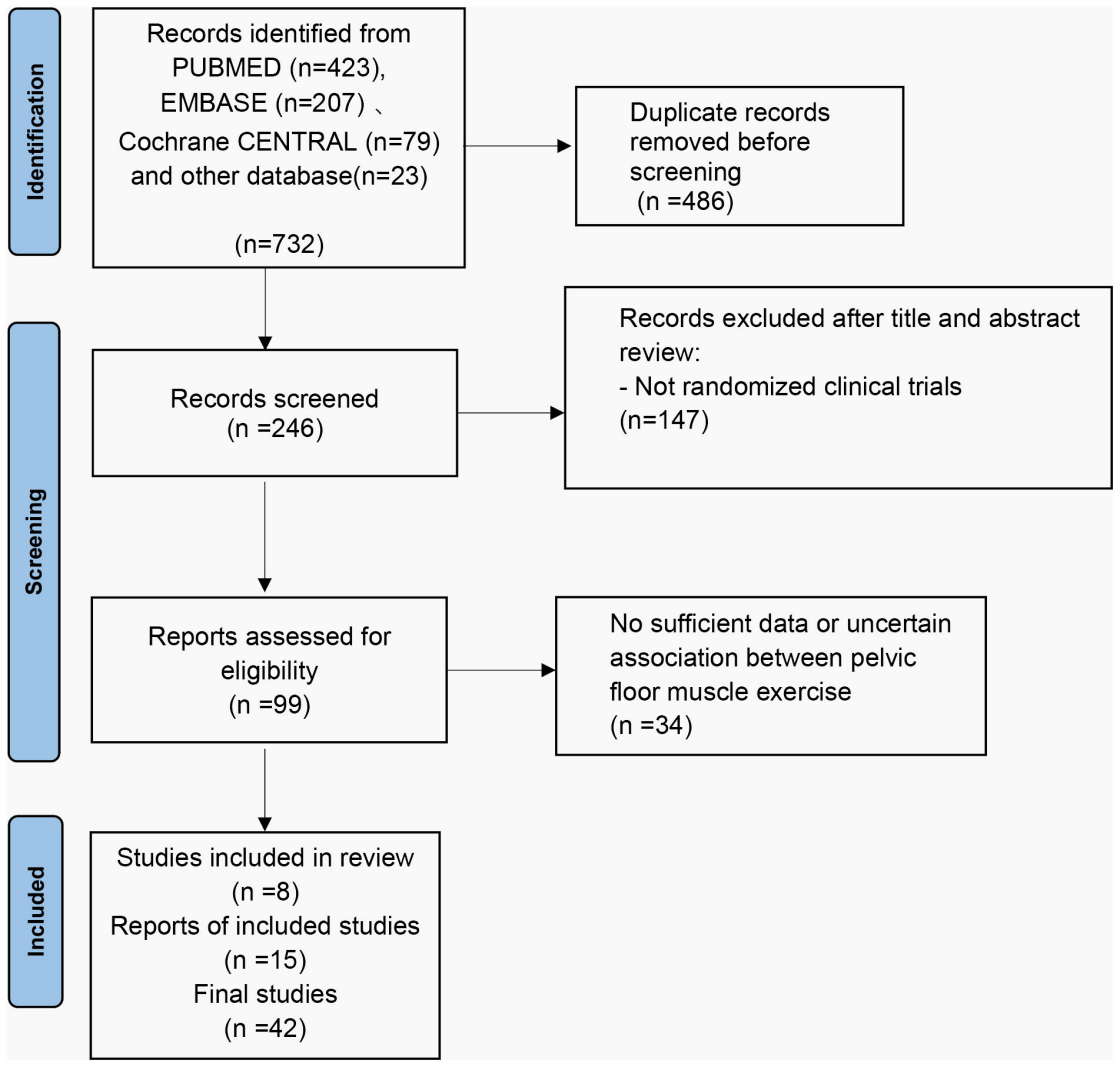
Literature screening process and results.

A total of 732 titles were obtained from the initial screening, including 423 titles from PubMed, 207 titles from EMBASE, 79 titles from Cochrane, 23 titles from other databases, and some conference papers. A total of 246 titles were obtained after de-duplication into Endnote literature management software, and 42 titles were included after initial screening and rescreening (**Figure 2**).

### 3.2 Basic characteristics of the included studies: risk of bias evaluation

A total of 4,256 subjects, 2,216 in the experimental group and 2,040 in the control group, were included in the 42 (8–49) RCTs included in this network meta-analysis, as shown in **Supplementary Table 1**. Among the interventions in the experimental group were Kegel exercises guided by a professional such as a physical instructor or nurse, bioelectric therapy, pharmacotherapy, biofeedback, and one or a combination of one or more of conventional pelvic floor muscle therapy, and, in the control group a combination of conventional care, one or more of conventional pelvic floor muscle training and electrotherapy. Conventional care includes conventional care of patients’ urethral orifice, change of urinary tube, and cleaning of perineum. Professional therapists and nurses include those with experience in pelvic floor exercises. The observed outcome indicators broadly describe the recovery of urinary control in patients after the different treatment modalities interventions and after different time periods in 1 month, 3 months, 6 months, and 12 months.

### 3.3 Risk of bias evaluation

**Figure 3:**
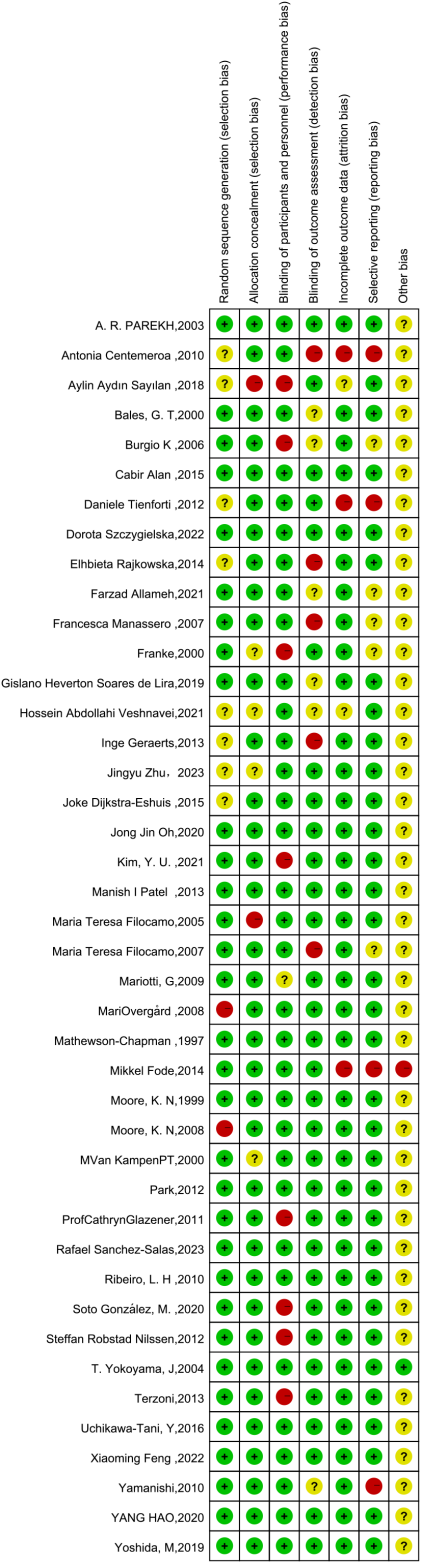
Literature bias evaluation results.

**Figure 4:**
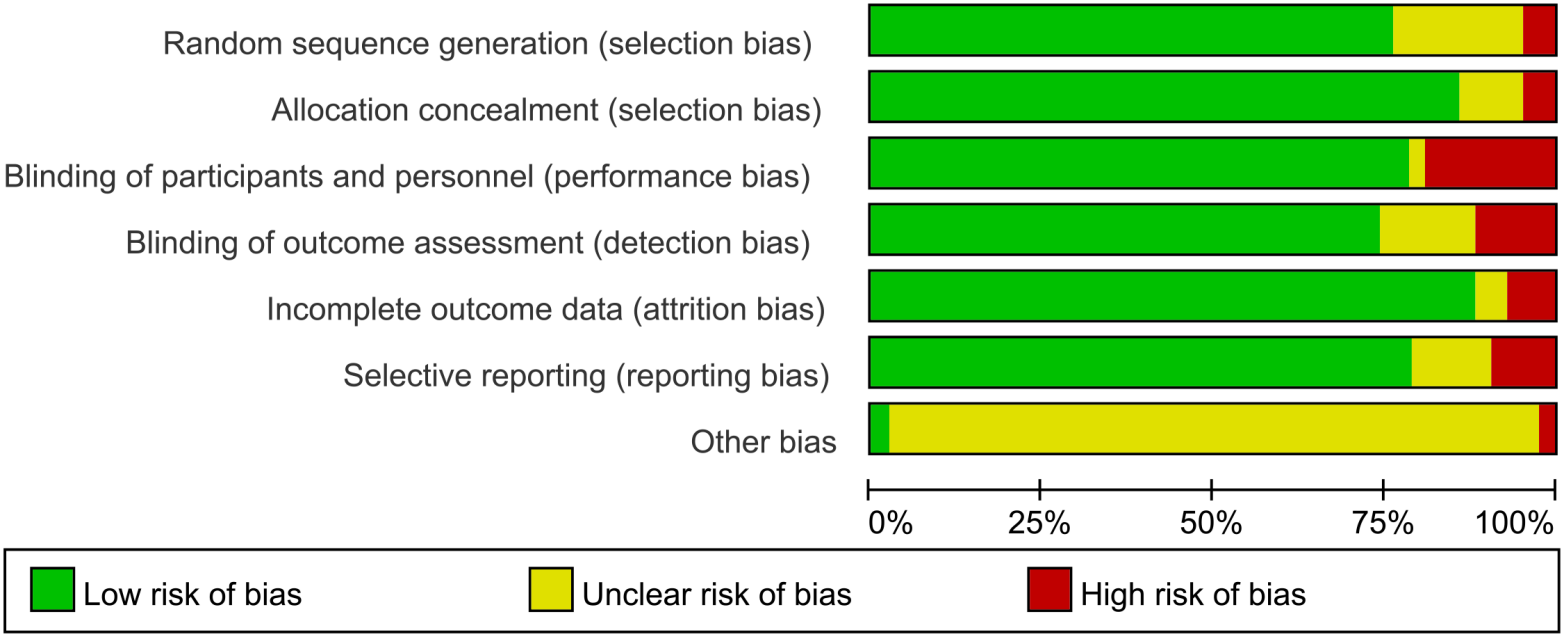
Results of literature bias evaluation.

This network meta-analysis was conducted using the Cochrane risk-of-bias assessment tool to assess the quality of the 42 included papers (see **Figures 3**, **4**, where red dots indicate a high risk of bias for each bias criterion, yellow states a moderate risk, and green indicates a low risk of bias). In a blinded assessment, if both the experimental and control groups had another form of training in addition to routine care, in both experimenter exchanges, patients may perceive themselves as better able to cooperate with treatment for the experimental group because of the additional training for both, and it has less psychological impact on patients, at which point such cases are identified as low risk of bias in the blinded bias assessment and, conversely, high risk of bias. Thirty-four studies specified a specific randomization scheme, with the generation of the randomized sequence being not specified in eight studies and with a high risk of bias being grouped in two studies. Of the publication bias, four studies were considered to be at high risk of bias, possibly due to their association with novel device development. Other sources of bias were judged to be unclear except for one study that may have a potential link to a medical device company or a company related to a novel treatment. One study clarified the absence of corporate sponsorship (**Figures 3**, **4**).

### 3.4 Results of the network meta-analysis

#### 3.4.1 The web of relationships

**Figure 5:**
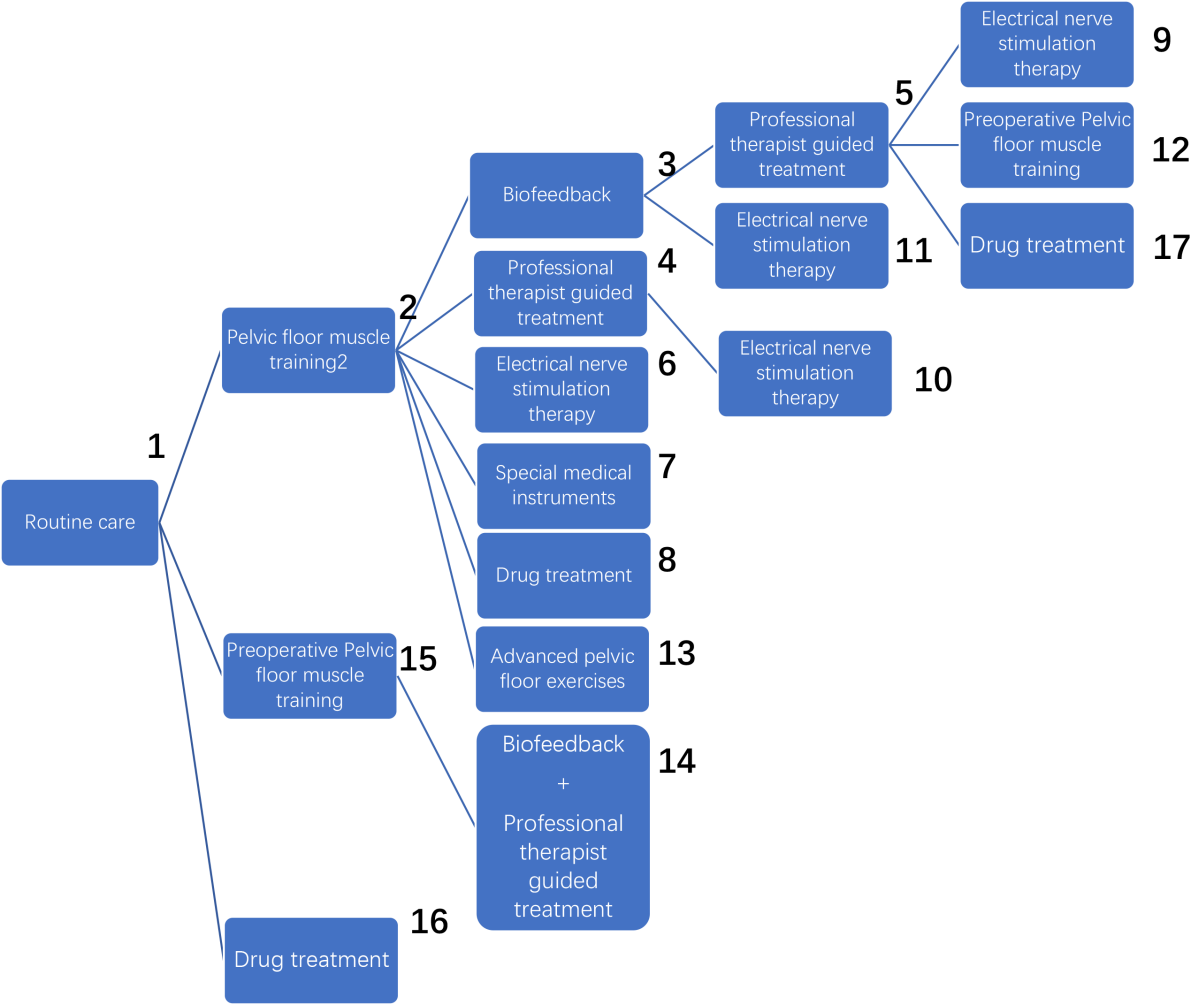
Treatment methods.

According to the order of the various treatment methods, the 17 treatment methods were classified as follows: routine care (1), routine care + pelvic floor muscle training (2), pelvic floor muscle training + routine care + biofeedback (3), routine care + pelvic floor muscle training + professional therapist–guided treatment (4), pelvic floor muscle training + routine care + biofeedback + professional therapist–guided treatment (5), pelvic floor muscle training + electrical nerve stimulation therapy + routine care (6), special medical instruments (penile vibratory stimulation (PVS) units) + routine care + pelvic floor muscle training (7), routine care + pelvic floor muscle training + drug treatment (duloxetine) (8), pelvic floor muscle training + routine care + biofeedback + professional therapist–guided treatment + electrical nerve stimulation therapy (9), routine care + electrical nerve stimulation therapy + professional therapist–guided treatment + pelvic floor muscle training (10), electrical nerve stimulation therapy + biofeedback + pelvic floor muscle training + routine care floor muscle training + routine care (11), pelvic floor muscle training + routine care + biofeedback + professional therapist–guided treatment + preoperative pelvic floor muscle training + routine care (12), pelvic floor muscle training and routine care + pelvic floor muscle training + advanced pelvic floor exercises (13), routine care + preoperative pelvic floor muscle training + biofeedback + professional therapist–guided (14), routine care + preoperative pelvic floor muscle training (15), routine care + drug treatment (duloxetine) (16), routine care + drug treatment (duloxetine) + pelvic floor muscle training + biofeedback + professional therapist–guided treatment (17), as shown in **Table 1** and **Figure 5**.

**Table 1:** Pelvic floor muscle treatment methods and corresponding numbers.

Treatment 1	Routine care
Treatment 2	Routine care + pelvic floor muscle training
Treatment 3	Pelvic floor muscle training + routine care + biofeedback
Treatment 4	Routine care + pelvic floor muscle training + professional therapist guided treatment
Treatment 5	Pelvic floor muscle training + routine care + biofeedback + professional therapist–guided treatment
Treatment 6	Pelvic floor muscle training + electrical nerve stimulation therapy + routine care
Treatment 7	Special medical instruments (PVS units) + routine care + pelvic floor muscle training
Treatment 8	Routine care + pelvic floor muscle training + drug treatment (duloxetine)
Treatment 9	Pelvic floor muscle training + routine care + biofeedback + professional therapist–guided treatment + electrical nerve stimulation therapy
Treatment 10	Routine care + electrical nerve stimulation therapy + professional therapist–guided treatment + pelvic floor muscle training
Treatment 11	Electrical nerve stimulation therapy + biofeedback + pelvic floor muscle training + routine care
Treatment 12	Pelvic floor muscle training + routine care + biofeedback + professional therapist–guided treatment + preoperative pelvic floor muscle training
Treatment 13	Routine care + pelvic floor muscle training + advanced pelvic floor exercises
Treatment 14	Routine care + preoperative pelvic floor muscle training + biofeedback + professional therapist–guided treatment
Treatment 15	Routine care + preoperative pelvic floor muscle training
Treatment 16	Routine care + drug treatment (duloxetine)
Treatment 17	Routine care + drug treatment (duloxetine) + pelvic floor muscle training + biofeedback + professional therapist–guided treatment

**Figure 6:**
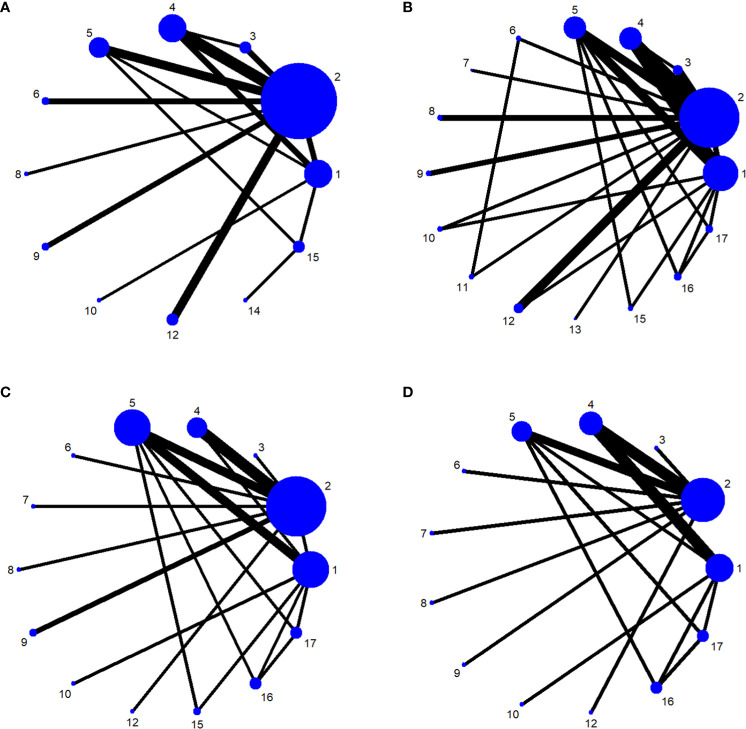
**(A)** One-month network chart. **(B)** Three-month network chart. **(C)** Six-month network chart. **(D)** Twelve-month network chart.

The network relationships for the treatments used to restore postoperative incontinence in patients undergoing radical prostatectomy are shown in **Figures 6A–D**. The network diagram represents the number of studies, with the thicker the line segment, the more studies are included; the circular area in the network diagram represents the sample size of the population using the measure, with the larger the circle, the larger the population included in the study. The line segments between the dots represent studies for which they are directly comparable.

#### 3.4.2 Inconsistency test

##### 3.4.2.1 Overall inconsistency test

The results of the overall inconsistency analysis showed that the overall effective rate was greater than 0.05, indicating that the results of direct and indirect comparisons were consistent across treatment modalities.

##### 3.4.2.2 Ring inconsistency test

The lower 95% CI for all closed-loop IFs involved in the indicator did not reach 0, suggesting that the loop inconsistency was statistically significant, whereas the rest of the loops were not statistically significant.

#### 3.4.3 Results of the network meta-analysis

##### 3.4.3.1 Total clinical effectiveness in 1 month

**Figure 7:**
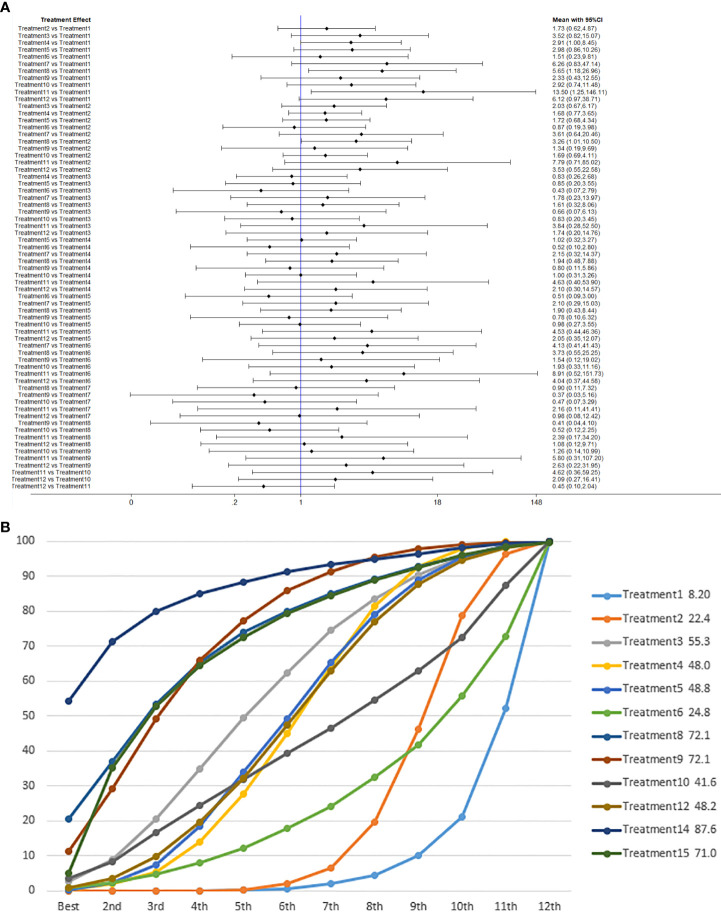
**(A)** The forest plot represents the OR and 95% CrI for a two-by-two comparison of multiple treatment modalities for urinary incontinence at 1 month after radical prostatectomy. **(B)** Cumulative probability graph of postoperative urinary incontinence recovered in 1 month.

We used network meta-analysis of different pelvic floor muscle treatment measures to assess the recovery of urinary incontinence in patients at 1 month after radical prostate cancer surgery. Treatment 9 (pelvic floor muscle training + routine care + biofeedback + professional therapist–guided treatment + electrical nerve) demonstrated better outcomes compared with treatment 1 (routine care) and treatment 2 (routine care + pelvic floor muscle training) at 1 month (OR: 5.65, 95% Confidence Range Interval (Crl): 1.18–26.96; OR: 3.26, 95% CrI: 1.01–10.50). Treatment 13 showed better results compared with treatment 1 at 1 month postoperatively (OR: 13.50, 95% CrI: 1.25–146.11), as shown in **Figure 7A**. The SUCRA values for the various treatments are shown in **Figure 7B**.

In **Figure 7B**, we can see that treatment 14 (routine care + preoperative pelvic floor muscle training + biofeedback + professional therapist–guided treatment), treatment 9 (pelvic floor muscle training + routine care + biofeedback + professional therapist–guided treatment + electrical nerve), and treatment 8 (routine care + pelvic floor muscle training + drug treatment (duloxetine) are ranked as the top three in terms of efficacy. In the network meta-analysis ladder table of urinary incontinence recovered within 1 month after surgery, we found that biofeedback therapy under professional guidance had a better therapeutic effect, and the drug duloxetine also had a better adjunctive effect on the recovery of postoperative incontinence in patients.

In the recovery of urinary incontinence after radical prostate cancer surgery, in **Table 2** we found the role of biofeedback therapy under professional guidance is evident during the first month of treatment, whereas drug treatment also plays a significant role in postoperative recovery. This means that, for a rapid recovery or improved control of incontinence, professionally supervised biofeedback pelvic floor muscle training supplemented with medication is required.

**Table 2:** Network meta-analysis ladder table of postoperative urinary incontinence recovered in 1 month.

Treatment 1											
0.58 (0.21, 1.62)	Treatment 2										
0.28 (0.07, 1.22)	0.49 (0.16, 1.50)	Treatment 3									
0.34 (0.12, 1.00)	0.60 (0.27, 1.29)	1.21 (0.37, 3.91)	Treatment 4								
0.34 (0.10, 1.16)	0.58 (0.23, 1.47)	1.18 (0.28, 4.95)	0.98 (0.31, 3.13)	Treatment 5							
0.66 (0.10, 4.28)	1.14 (0.25, 5.22)	2.32 (0.36, 15.03)	1.92 (0.36, 10.36)	1.97 (0.33, 11.61)	Treatment 6						
0.16 (0.02, 1.20)	0.28 (0.05, 1.57)	0.56 (0.07, 4.42)	0.47 (0.07, 3.12)	0.48 (0.07, 3.41)	0.24 (0.02, 2.43)	Treatment 8					
0.18 (0.04, 0.84)	0.31 (0.10, 0.99)	0.62 (0.12, 3.12)	0.52 (0.13, 2.09)	0.53 (0.12, 2.34)	0.27 (0.04, 1.81)	1.11 (0.14, 8.97)	Treatment 9				
0.43 (0.08, 2.31)	0.74 (0.10, 5.37)	1.51 (0.16, 13.99)	1.25 (0.17, 9.18)	1.28 (0.16, 10.34)	0.65 (0.05, 8.05)	2.69 (0.19, 37.27)	2.43 (0.24, 24.15)	Treatment 10			
0.34 (0.09, 1.34)	0.59 (0.24, 1.45)	1.20 (0.29, 5.00)	1.00 (0.31, 3.24)	1.02 (0.28, 3.69)	0.52 (0.09, 3.00)	2.14 (0.30, 15.06)	1.93 (0.44, 8.41)	0.80 (0.09, 6.97)	Treatment 12		
0.07 (0.01, 0.80)	0.13 (0.01, 1.40)	0.26 (0.02, 3.56)	0.22 (0.02, 2.51)	0.22 (0.02, 2.26)	0.11 (0.01, 1.91)	0.46 (0.02, 8.89)	0.42 (0.03, 5.99)	0.17 (0.01, 3.19)	0.22 (0.02, 2.78)	Treatment 14	
0.16 (0.03, 1.03)	0.28 (0.04, 1.81)	0.57 (0.07, 4.88)	0.48 (0.07, 3.30)	0.49 (0.08, 2.86)	0.25 (0.02, 2.73)	1.02 (0.08, 12.98)	0.92 (0.10, 8.28)	0.38 (0.03, 4.63)	0.48 (0.06, 3.75)	2.21 (0.49, 9.95)	Treatment 15

##### 3.4.3.2 Total clinical effectiveness in 3 months

We used a network meta-analysis of different pelvic floor muscle treatment measures to assess the recovery of urinary incontinence in patients at 3 months after radical prostate cancer surgery.

Treatment 6 (pelvic floor muscle training + electrical nerve stimulation therapy + routine care) showed better results at 3 months compared with treatment 1 (routine care), treatment 2 (routine care + pelvic floor muscle training), treatment 4 (routine care + pelvic floor muscle training + professional therapist–guided treatment), treatment 5 (pelvic floor muscle training + routine care + biofeedback + professional therapist–guided treatment), treatment 7 [special medical instruments (PVS units) + routine care + pelvic floor muscle training], treatment 12 (pelvic floor muscle training + routine care + biofeedback + professional therapist–guided treatment + preoperative pelvic), treatment 16 [routine care + drug treatment (duloxetine)], and treatment 17 [routine care + drug treatment (duloxetine) + pelvic floor muscle training + biofeedback + professional therapist–guided treatment] at 3 months postoperatively (OR: 18.06, 95% CrI: 3.00–108.71; OR: 16.40, 95% CrI: 3.22–83.57; OR: 10.81, 95% CrI: 1.83–63.97; OR: 9.35, 95% CrI: 1.47–59.38; OR: 14.05, 95% CrI: 1.24–159.37; OR: 11.76, 95% CrI: 1.85–74.73; OR: 18.85, 95% CrI: 1.85–192.29; OR: 34.02, 95% CrI: 3.07–377.25). Treatment 9 (pelvic floor muscle training + routine care + biofeedback + professional therapist–guided treatment + electrical nerve) showed better results compared with treatment 1 (routine care), treatment 2 (routine care + pelvic floor muscle training), treatment 4 (routine care + pelvic floor muscle training + professional therapist–guided treatment), treatment 12 (pelvic floor muscle training + routine care + biofeedback + professional therapist–guided treatment + preoperative pelvic), treatment 16 [routine care + drug treatment (duloxetine)], and treatment 17 [routine care + drug treatment (duloxetine) + pelvic floor muscle training + biofeedback + professional therapist–guided treatment] (OR: 7.89, 95% CrI: 1.89–32.96; OR: 7.16, 95% CrI: 2.13–24.12; OR: 4.72, 95% CrI: 1.16–19.31; OR: 5.14, 95% CrI: 1.15–22.96). Treatment 11 (electrical nerve stimulation therapy + biofeedback + pelvic floor muscle training + routine care) showed better results compared with treatment 17 [routine care + drug treatment (duloxetine) + pelvic floor muscle training + biofeedback + professional therapist–guided treatment] (OR: 13.76, 95% CrI: 1.17–161.77).

**Figure 8:**
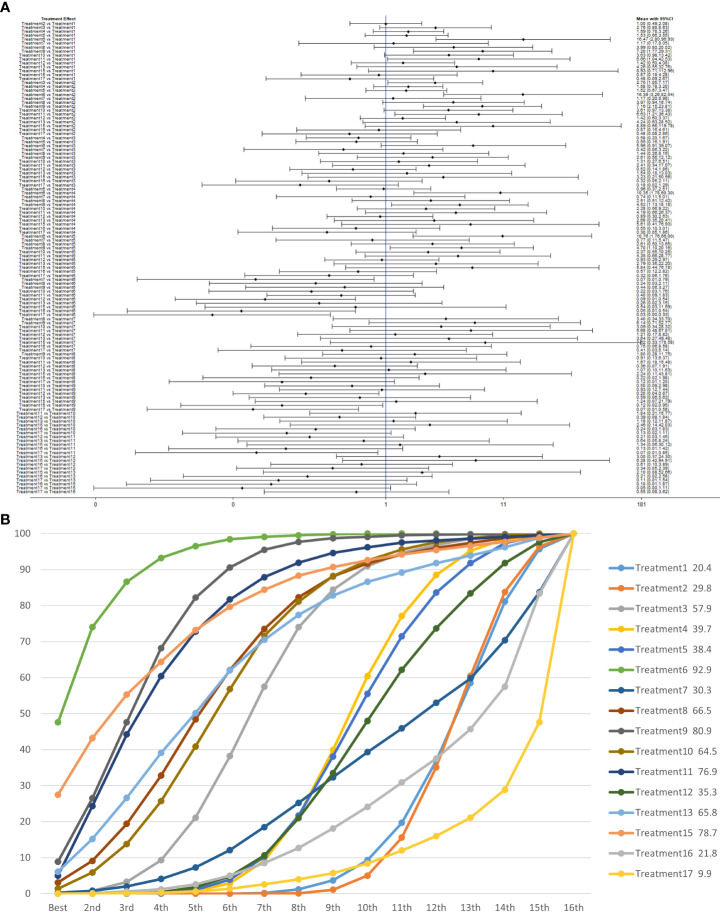
**(A)** The forest plot represents the OR and 95% CrI for a two-by-two comparison of multiple treatment modalities for urinary incontinence at 3 months after radical prostatectomy. **(B)** Cumulative probability graph of postoperative urinary incontinence recovered in 3 months.

Treatment 3 (pelvic floor muscle training + routine care + biofeedback) showed better results compared with treatment 2 (routine care + pelvic floor muscle training) (OR: 2.71, 95% CrI: 1.03–7.14). Treatment 10 (routine care + electrical nerve stimulation therapy + professional therapist–guided treatment + pelvic floor muscle) showed better results compared with treatment 1 (routine care) (OR: 3.80, 95% CrI: 1.01–14.26). Treatment 11 (electrical nerve stimulation therapy + biofeedback + pelvic floor muscle training + routine care) showed better results compared with treatment 1 (routine care) and treatment 2 (routine care + pelvic floor muscle training) (OR: 7.32, 95% CrI: 1.12–47.80; OR: 6.65, 95% CrI: 1.20–36.97) at 3 months postoperatively, as shown in **Figure 8A**. The SUCRA values for the various treatments are shown in **Figure 8B**.

In **Figure 8B**, we can see that treatment 6 (pelvic floor muscle training + electrical nerve stimulation therapy + routine care), treatment 9 (pelvic floor muscle training + routine care + biofeedback + professional therapist–guided treatment + electrical nerve), and treatment 15 (routine care + preoperative pelvic floor muscle training) are in the top three in terms of effectiveness. In the network meta-analysis ladder table of postoperative urinary incontinence recovered in 3 months, we could find that treatment 6 (pelvic floor muscle training + electrical nerve stimulation therapy + routine care) was better than most of the treatments at 3 months.

In the short to medium term, in **Table 3** we found electrostimulation has been shown to have a significant effect on the recovery of urinaryincontinence, and this has led to the need for more electrical stimulation of the relevant pelvic floor nerves. Similarly, biofeedback is also useful in the short to medium term. In **Table 4**, we found that preoperative pelvic floor muscle exercise may affect the effectiveness of electrotherapy for postoperative urinary incontinence in patients and that preoperative pelvic floor muscle training is not a good option for patients who want to treat urinary incontinence with electrotherapy after radical prostate cancer surgery.

**Table 3:** Network meta-analysis ladder table of postoperative urinary incontinence recovered in 6 months.

Treatment 1													
1.85 (0.57, 6.00)	Treatment 2												
0.03 (0.00, 0.94)	0.01 (0.00, 0.42)	Treatment 3											
0.64 (0.16, 2.48)	0.34 (0.12, 0.96)	23.61 (0.71, 790.05)	Treatment 4										
1.26 (0.43, 3.65)	0.68 (0.23, 2.02)	46.62 (1.37, 1587.93)	1.97 (0.49, 7.91)	Treatment 5									
0.48 (0.04, 5.24)	0.26 (0.03, 2.08)	17.72 (0.34, 922.15)	0.75 (0.07, 7.72)	0.38 (0.04, 4.02)	Treatment 6								
4.33 (0.32, 58.80)	2.33 (0.23, 23.99)	160.68 (2.70, 9548.99)	6.81 (0.53, 87.11)	3.45 (0.26, 45.21)	9.07 (0.40, 207.32)	Treatment 7							
0.34 (0.03, 3.78)	0.18 (0.02, 1.50)	12.52 (0.24, 660.18)	0.53 (0.05, 5.58)	0.27 (0.02, 2.90)	0.71 (0.04, 13.80)	0.08 (0.00, 1.81)	Treatment 8						
0.34 (0.05, 2.18)	0.18 (0.04, 0.78)	12.58 (0.33, 485.49)	0.53 (0.09, 3.18)	0.27 (0.04, 1.66)	0.71 (0.06, 9.01)	0.08 (0.01, 1.22)	1.00 (0.08, 13.01)	Treatment 9					
0.23 (0.03, 2.00)	0.12 (0.01, 1.45)	8.57 (0.13, 548.00)	0.36 (0.03, 4.66)	0.18 (0.02, 2.04)	0.48 (0.02, 12.16)	0.05 (0.00, 1.58)	0.68 (0.03, 17.48)	0.68 (0.04, 11.79)	Treatment 10				
1.85 (0.16, 22.05)	1.00 (0.11, 8.84)	68.87 (1.26, 3762.83)	2.92 (0.26, 32.56)	1.48 (0.13, 16.92)	3.89 (0.19, 79.56)	0.43 (0.02, 10.42)	5.50 (0.26, 114.53)	5.48 (0.40, 74.91)	8.03 (0.30, 214.38)	Treatment 12			
0.50 (0.03, 8.17)	0.27 (0.01, 5.16)	18.67 (0.21, 1622.82)	0.79 (0.04, 16.70)	0.40 (0.02, 6.92)	1.05 (0.03, 39.01)	0.12 (0.00, 4.97)	1.49 (0.04, 56.01)	1.48 (0.06, 39.47)	2.18 (0.06, 73.99)	0.27 (0.01, 10.59)	Treatment 15		
1.48 (0.25, 8.64)	0.80 (0.11, 5.65)	54.95 (1.13, 2671.06)	2.33 (0.28, 19.11)	1.18 (0.20, 6.88)	3.10 (0.18, 54.27)	0.34 (0.02, 7.17)	4.39 (0.25, 78.20)	4.37 (0.38, 49.83)	6.41 (0.39, 104.11)	0.80 (0.04, 14.93)	2.94 (0.12, 75.03)	Treatment 16	
2.27 (0.38, 13.46)	1.22 (0.17, 8.78)	84.19 (1.72, 4123.43)	3.57 (0.43, 29.69)	1.81 (0.30, 10.72)	4.75 (0.27, 84.00)	0.52 (0.02, 11.09)	6.72 (0.37, 121.03)	6.69 (0.58, 77.26)	9.82 (0.60, 161.19)	1.22 (0.06, 23.11)	4.51 (0.18, 115.99)	1.53 (0.22, 10.84)	Treatment 17

**Table 4:** Network meta-analysis ladder table of postoperative urinary incontinence recovered in 3 months.

Treatment 1															
1.00 (0.48, 2.06)	Treatment 2														
0.36 (0.12, 1.13)	0.36 (0.14, 0.95)	Treatment 3													
0.63 (0.31, 1.29)	0.63 (0.31, 1.27)	1.74 (0.60, 5.02)	Treatment 4												
0.65 (0.28, 1.52)	0.66 (0.29, 1.50)	1.80 (0.52, 6.22)	1.04 (0.40, 2.72)	Treatment 5											
0.06 (0.01, 0.36)	0.06 (0.01, 0.31)	0.17 (0.03, 1.10)	0.10 (0.02, 0.56)	0.09 (0.02, 0.57)	Treatment 6										
0.85 (0.12, 5.86)	0.86 (0.14, 5.11)	2.36 (0.31, 17.87)	1.36 (0.20, 9.24)	1.31 (0.18, 9.32)	14.05 (1.26, 156.12)	Treatment 7									
0.25 (0.05, 1.26)	0.25 (0.06, 1.06)	0.69 (0.12, 3.91)	0.40 (0.08, 1.97)	0.38 (0.07, 2.01)	4.13 (0.47, 35.93)	0.29 (0.03, 2.91)	Treatment 8								
0.14 (0.03, 0.57)	0.14 (0.04, 0.46)	0.38 (0.08, 1.79)	0.22 (0.06, 0.89)	0.21 (0.05, 0.91)	2.29 (0.31, 17.15)	0.16 (0.02, 1.40)	0.55 (0.09, 3.61)	Treatment 9							
0.28 (0.07, 1.02)	0.28 (0.07, 1.03)	0.76 (0.15, 3.77)	0.44 (0.11, 1.78)	0.42 (0.10, 1.82)	4.54 (0.57, 36.38)	0.32 (0.04, 2.96)	1.10 (0.16, 7.70)	1.98 (0.34, 11.74)	Treatment 10						
0.15 (0.02, 0.96)	0.15 (0.03, 0.83)	0.41 (0.06, 2.93)	0.24 (0.04, 1.50)	0.23 (0.03, 1.52)	2.47 (0.55, 11.20)	0.18 (0.01, 2.07)	0.60 (0.06, 5.55)	1.08 (0.13, 8.68)	0.54 (0.06, 4.67)	Treatment 11					
0.70 (0.25, 2.01)	0.71 (0.30, 1.68)	1.94 (0.54, 7.00)	1.12 (0.38, 3.29)	1.08 (0.34, 3.44)	11.58 (1.85, 72.47)	0.82 (0.11, 5.99)	2.80 (0.52, 15.05)	5.06 (1.15, 22.25)	2.55 (0.54, 11.93)	4.68 (0.69, 31.70)	Treatment 12				
0.23 (0.03, 1.80)	0.24 (0.04, 1.58)	0.65 (0.08, 5.47)	0.37 (0.05, 2.84)	0.36 (0.05, 2.86)	3.86 (0.32, 47.01)	0.28 (0.02, 3.74)	0.94 (0.09, 10.19)	1.69 (0.18, 16.06)	0.85 (0.08, 8.59)	1.56 (0.12, 20.14)	0.33 (0.04, 2.71)	Treatment 13			
0.11 (0.01, 1.42)	0.11 (0.01, 1.52)	0.31 (0.02, 4.83)	0.18 (0.01, 2.44)	0.17 (0.01, 2.25)	1.84 (0.09, 39.42)	0.13 (0.01, 3.08)	0.45 (0.02, 8.74)	0.81 (0.05, 14.14)	0.41 (0.02, 6.93)	0.75 (0.03, 16.75)	0.16 (0.01, 2.41)	0.48 (0.02, 11.99)	Treatment 15		
1.14 (0.23, 5.61)	1.15 (0.22, 6.10)	3.16 (0.47, 21.03)	1.82 (0.33, 9.98)	1.75 (0.36, 8.63)	18.85 (1.85, 192.29)	1.34 (0.12, 15.43)	4.57 (0.50, 41.30)	8.24 (1.05, 64.31)	4.15 (0.55, 31.46)	7.62 (0.70, 82.63)	1.63 (0.26, 10.31)	4.88 (0.39, 61.38)	10.22 (0.53, 195.83)	Treatment 16	
2.07 (0.37, 11.41)	2.08 (0.35, 12.33)	5.70 (0.77, 42.01)	3.29 (0.54, 20.15)	3.16 (0.57, 17.55)	34.02 (3.07, 377.25)	2.42 (0.19, 30.16)	8.24 (0.83, 81.37)	14.86 (1.73, 127.49)	7.49 (0.90, 62.45)	13.76 (1.17, 161.77)	2.94 (0.42, 20.65)	8.80 (0.65, 119.61)	18.45 (0.90, 377.58)	1.80 (0.28, 11.80)	Treatment 17

##### 3.4.3.3 Total clinical effectiveness in 6 months

We used a network meta-analysis of different pelvic floor muscle treatment measures to assess the recovery of urinary incontinence in patients at 6 months after radical prostate cancer surgery.

**Figure 9:**
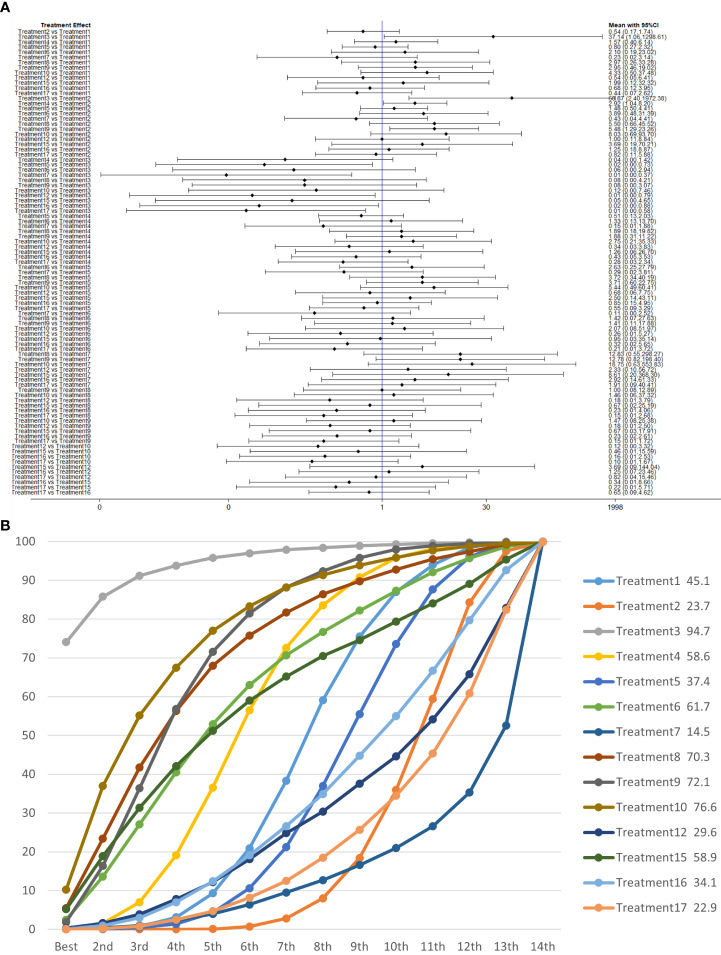
**(A)** The forest plot represents the OR and 95% CrI for a two-by-two comparison of multiple treatment modalities for urinary incontinence at 6 months after radical prostatectomy. **(B)** Cumulative probability graph of postoperative urinary incontinence recovered in 6 months.

Treatment 3 (pelvic floor muscle training + routine care + biofeedback) showed better results compared with treatment 1 (routine care), treatment 2 (routine care + pelvic floor muscle training), treatment 5 (pelvic floor muscle training + routine care + biofeedback + professional therapist–guided treatment), treatment 7 [special medical instruments (PVS units) + routine care + pelvic floor muscle training], treatment 12 (pelvic floor muscle training + routine care + biofeedback + professional therapist–guided treatment + preoperative pelvic floor muscle training), treatment 16 [routine care + drug treatment (duloxetine), and treatment 17 [routine care + drug treatment (duloxetine) + pelvic floor muscle training + biofeedback + professional therapist–guided treatment] (OR: 43.43, 95% CrI: 1.15–1,642.47; OR: 68.87, 95% CrI: 2.30–2,060.60; OR: 46.62, 95% CrI: 1.37–1,587.93; OR: 160.68, 95% CrI: 2.70–9,548.99; OR: 68.87, 95% CrI: 1.26–3,762.83; OR: 54.95, 95% CrI: 1.13–2,671.06; OR: 84.19, 95% CrI: 1.13–2,671.06). Treatment 2 (routine care + pelvic floor muscle training) showed worse results compared with treatment 4 (routine care + pelvic floor muscle training + professional therapist–guided treatment) and treatment 9 (pelvic floor muscle training + routine care + biofeedback + professional therapist–guided treatment + electrical nerve) (OR: 0.34, 95% CrI: 0.12–0.96; OR: 0.18, 95% CrI: 0.04–0.78) at 6 months postoperatively, as shown in **Figure 9A**. The SUCRA values for the various treatments are shown in **Figure 9B**.

In **Figure 9B**, we can see that treatment 3 (pelvic floor muscle training + routine care + biofeedback), treatment 10 (routine care + electrical nerve stimulation therapy + professional therapist–guided treatment + pelvic floor muscle), and treatment 9 (pelvic floor muscle training + routine care + biofeedback + professional therapist–guided treatment + electrical nerve) are in the top three in terms of effectiveness. In the network meta-analysis ladder table of postoperative urinary incontinence recovered in 6 months, we could find that treatment 3 (pelvic floor muscle training + routine care + biofeedback) was better than treatment 5 (pelvic floor muscle training + routine care + biofeedback + professional therapist–guided treatment) and treatment 12 (pelvic floor muscle training + routine care + biofeedback + professional therapist–guided treatment + preoperative pelvic).

In **Table 5**, we found that electrical stimulation and biofeedback therapy remained effective during the 6-month interim treatment period. At the 6-month interim treatment, preoperative pelvic floor muscle training still had a detrimental effect on the postoperative electrical stimulation treatment and, unexpectedly, on the guided treatment by the specialist therapist, which may be related to the level of the specialist therapist.

**Table 5:** Network meta-analysis ladder table of postoperative urinary incontinence recovered in 12 months.

Treatment 1												
1.31 (0.36, 4.78)	Treatment 2											
0.11 (0.00, 3.81)	0.08 (0.00, 2.28)	Treatment 3										
0.55 (0.21, 1.47)	0.42 (0.15, 1.21)	5.14 (0.16, 167.61)	Treatment 4									
1.24 (0.31, 4.93)	0.95 (0.27, 3.37)	11.55 (0.33, 404.45)	2.25 (0.56, 9.06)	Treatment 5								
0.63 (0.06, 6.46)	0.48 (0.07, 3.33)	5.86 (0.13, 273.67)	1.14 (0.13, 10.29)	0.51 (0.05, 5.12)	Treatment 6							
2.62 (0.17, 41.18)	2.00 (0.18, 22.74)	24.35 (0.40, 1493.55)	4.74 (0.34, 66.95)	2.11 (0.14, 32.71)	4.15 (0.19, 92.62)	Treatment 7						
0.32 (0.02, 6.58)	0.24 (0.02, 3.76)	2.93 (0.04, 218.45)	0.57 (0.03, 10.81)	0.25 (0.01, 5.23)	0.50 (0.02, 14.37)	0.12 (0.00, 4.72)	Treatment 8					
0.13 (0.01, 1.61)	0.10 (0.01, 0.86)	1.21 (0.02, 63.31)	0.23 (0.02, 2.58)	0.10 (0.01, 1.27)	0.21 (0.01, 3.72)	0.05 (0.00, 1.28)	0.41 (0.01, 13.54)	Treatment 9				
0.09 (0.01, 1.25)	0.07 (0.00, 1.28)	0.82 (0.01, 69.64)	0.16 (0.01, 2.68)	0.07 (0.00, 1.40)	0.14 (0.00, 4.74)	0.03 (0.00, 1.54)	0.28 (0.01, 15.74)	0.68 (0.02, 26.19)	Treatment 10			
0.64 (0.04, 9.20)	0.49 (0.05, 5.02)	5.94 (0.10, 343.82)	1.16 (0.09, 14.91)	0.51 (0.04, 7.31)	1.01 (0.05, 20.91)	0.24 (0.01, 7.08)	2.03 (0.06, 74.45)	4.92 (0.21, 117.87)	7.20 (0.17, 306.99)	Treatment 12		
2.11 (0.39, 11.38)	1.61 (0.25, 10.41)	19.55 (0.43, 884.00)	3.80 (0.61, 23.56)	1.69 (0.32, 8.93)	3.33 (0.23, 48.99)	0.80 (0.04, 17.23)	6.67 (0.24, 185.09)	16.20 (0.93, 281.04)	23.71 (1.03, 544.32)	3.29 (0.17, 65.30)	Treatment 16	
1.96 (0.36, 10.71)	1.50 (0.23, 9.78)	18.21 (0.40, 827.30)	3.54 (0.57, 22.16)	1.58 (0.30, 8.40)	3.11 (0.21, 45.93)	0.75 (0.03, 16.14)	6.21 (0.22, 173.33)	15.10 (0.87, 263.41)	22.09 (0.96, 509.90)	3.07 (0.15, 61.18)	0.93 (0.16, 5.39)	Treatment 17

### 3.5 Total clinical effectiveness at 12 months

**Figure 10:**
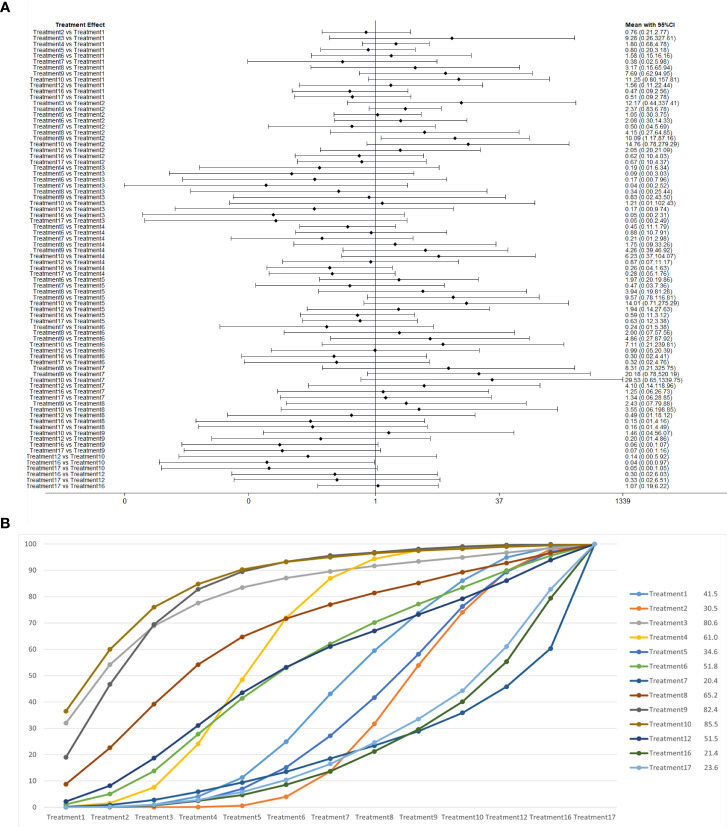
**(A)** The forest plot represents the OR and 95% CrI for a two-by-two comparison of multiple treatment modalities for urinary incontinence at 12 months after radical prostatectomy. **(B)** Cumulative probability graph of postoperative urinary incontinence recovered in 12 months.

We used a network meta-analysis of different pelvic floor muscle treatment measures to assess the recovery of urinary incontinence in patients at 12 months after radical prostate cancer surgery. Treatment 2 (routine care + pelvic floor muscle training) showed better results compared with treatment 9 (pelvic floor muscle training + routine care + biofeedback + professional therapist–guided treatment + electrical nerve) (OR: 0.10, 95% CrI: 0.01–0.86) at 12 months postoperatively, as shown in **Figure 10A**. The SUCRA values for the various treatments are shown in **Figure 10B**.

In **Figure 10B**, we can see that treatment 9, treatment 10, and treatment 3 are in the top three in terms of effectiveness. In the long term, comprehensive treatment remains at the top of the treatment effectiveness scale, whereas the rest of the treatment modalities do not produce significant changes, which may be consistent with previous research that various treatments help control incontinence in the short term, but have similar effects in the long term.

In general, biofeedback and pelvic floor training can be used to treat incontinence in the early post-operative period in order to maintain a better recovery, and, where possible, specialist treatment is also required. Professional treatment can also have a significant effect on the first and middle post-operative period, and it is advisable to also offer bioelectric stimulation in the post-operative period. In the longer term, there is no significant difference between the treatments, and, for those who are not financially able to do so, we can use general pelvic floor training, which is slightly less effective in the early stages of treatment, but, in the long term, the two treatments are similar. At the same time, we can also start to develop more advanced medical devices for the treatment.

#### 3.5.1 Small sample effect estimation

**Figure 11:**
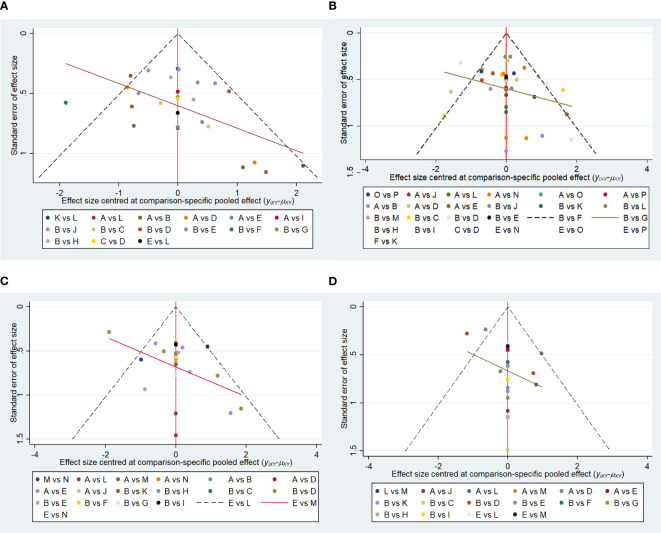
**(A)** Funnel plot of references cited in 1 month. **(B)** Funnel plot of references cited in 3 months. **(C)** Funnel plot of references cited in 6 months. **(D)** Funnel plot of references cited in 12 months.

Funnel plots were drawn for the total effective outcome indicator to test for publication bias. The results showed that all studies were generally symmetrically distributed around the X = 0 vertical line, and most studies fell inside the funnel, whereas some fell at the bottom, suggesting a possible small sample effect (**Figures 11A–D**).

There are some limitations in this study: all of the naive studies were in English, and most of them were of high quality in terms of allocation concealment and blinded implementation, but there may still be some bias. This suggests that future studies should pay attention to the reporting of safety.

This study has considerable merit in that the quality of the literature is relatively high and the included literature has a low publication bias. This network meta-analysis did not require a high level of gender, age, and basic physical fitness of the patients studied, and the relatively small amount of relevant foreign language literature retrieved so far could be used to increase the amount of data collected later in the study to better define the findings.

In summary, the results of this study showed that pelvic floor muscle training combined with biofeedback and guidance from professional therapists had a better recovery effect on urinary incontinence about 1 month after surgery than conventional care and pelvic floor muscle training. Bioelectrical stimulation combined with pelvic floor muscle exercise has a good recovery effect at 3 months; biofeedback treatment is more conducive to the recovery of urinary incontinence at 6 months after surgery; at 12 months, combined with biofeedback and electrical stimulation, therapist-guided pelvic floor muscle training is better than traditional pelvic floor muscle training.

## 4 Discussion

Urinary incontinence in individuals with radical prostate cancer primarily arises from structural or functional irregularities in the urethral sphincter. This can encompass damage to both the external urethral sphincter and associated nerves, as well as an inadequate length of the functional urethra. These issues subsequently induce alterations in the anatomy and function of the bladder and its outlet. Consequently, some patients may encounter urinary incontinence, attributed to diverse factors, including damage to the detrusor muscle, the duration of the extraction procedure, and individual variations in physical condition (50, 51).

“No pad” after radical prostatectomy is currently considered to have the best effect in assessing the effect of other factors on urinary incontinence (52). The baseline level (surgical method, degree of urinary incontinence, and other physical indicators) of patients after radical prostatectomy also has a certain impact on the treatment effect of patients. In terms of surgical methods, patients with robot-assisted radical prostatectomy recover the fastest after surgery (53); in surgical approach, perineal radical resection of prostate cancer is more effective than peritoneal radical resection; in surgical procedure, retention of lateral prostatic fascia (54), anterior and posterior fascia (55), nerve (56), and distal urethral sphincter complex (57) can accelerate the occurrence of postoperative urinary incontinence. In terms of physical indicators, younger patients (58), less weight (body mass index < 30) and frequent physical exercise (1 h or more per week) (59), and patients with longer preoperative membranous urethral length (60) showed faster recovery of postoperative urinary incontinence. However, the baseline level only had an impact on the early urinary incontinence back-resuscitation after radical prostatectomy, and there was no significant difference in the recovery of long-term urinary incontinence.

The common clinical treatment modalities include surgical treatment, and non-surgical treatment includes 1. pelvic floor muscle training (preoperative, postoperative, and ultrasound-guided), 2. electrical stimulation (electrical nerve stimulation - perineum, and electrotherapy - anal electrical stimulation), 3. lifestyle modification, 4. external penile compression devices, 5. conservative treatment (reducing coffee intake and weight loss), 6. medication (duloxetine, etc.), 7. endoscopic treatment, 8. urethral fillers (collagen injections), 9. specialist therapist supervision, 10. bladder training and surgical treatment, 11. sling surgery, and 12. the implantation of an artificial sphincter.

In our studies, we have found that the treatment or prevention of urinary incontinence through preoperative or postoperative measures has a significant effect on the development of complications of urinary incontinence in the short term but not in the long term. This does not mean that this range of therapeutic measures is not sufficiently effective, provided that we can reduce the duration of the effects of complications in a proactive way to bring about an improvement in the quality of life of patients after surgery and also to reduce the burden of disease on patients and health institutions (61). In terms of economic effects, biofeedback and professional therapist–guided treatment have similar treatment prices (62), and electrical nerve stimulation therapy is often more expensive than conventional care in the treatment of patients (63). Pelvic floor muscle therapy alone and usual care are more cost-effective than other forms of treatment. Taking into account quality adjusted life year, we point out that treatment for only severe life-affecting incontinence is likely to be cost-effective. We also state that, when severe urinary incontinence occurs after surgery, pelvic floor muscle exercise therapy such as biofeedback, electrical stimulation, and personal guidance can effectively increase the recovery of urinary incontinence in the first 3 months. When urinary incontinence does not have a great impact on life, conservative treatment and ordinary pelvic floor muscle exercise may be more cost-effective.

During pelvic floor exercises, which are difficult to assess and often have an impact on the management of pelvic floor exercises, relying on professional guidance and biofeedback can be burdensome to treatment. If the patient can be given this training preoperatively, it may be possible to reduce the cost and time and effort of treatment by allowing the patient to complete this modified version of the pelvic floor exercise, and, more fortunately, a new device has been investigated by Hodges and others, and we will soon see the results of the device (64, 65).

Validity testing of the stopwatch may be a valid assessment tool when determining the strength of the pelvic floor muscles exercised after radical prostate cancer surgery, in a simple test that can determine the degree of strength of the patient’s pelvic floor muscles, which may be a better option compared with complex electrophysiological activity tests.

In a study by Tantawy and others (66), whole-body vibration training has been shown to be effective as an alternative to traditional treatment for patients with post-radical prostate cancer incontinence (66). In a study by Centemero et al., pelvic floor training prior to surgery in the perioperative period for radical prostate cancer improved postoperative urinary incontinence (22).

When patients have different degrees of post-operative incontinence, different treatment modalities should be used. Conservative treatment including Endo urethral injection can be used for mild incontinence that has only a minor impact on life, whereas surgical treatment is more effective when the incontinence has reached a level that seriously affects the patient’s quality of life (67).

In a study of related drug treatments, it was found that, in addition to duloxetine, proviverrine hydrochloride, and vardenafil and tadalafil as phosphodiesterase type 5 inhibitors (PDE5-I), solifenacin as M-type choline receptor antagonist, solifenacin, is also effective in early postoperative urinary incontinence (68–71).

The use of PDE5-I improves the quality of life of patients after surgery and is associated with its ability to relieve urinary incontinence (72). Solifenacin’s effect is mainly to reduce the probability of postoperative complications by improving detrusor overactivity (DO) and cytometric capacity (73).

Non-surgical management of incontinence after radical prostate cancer surgery can also be managed by bladder training, penciled clamps, endoscopic therapy, injections, and lifestyle modifications such as improving lifestyle, reducing coffee, and weight loss, but little research has been reported on these modalities. It is hoped that more research will be conducted on these approaches in future studies (74). Moreover, families with high medical burdens can wait for the natural recovery of incontinence instead of using more costly alternative treatments. As more relevant trials are conducted, in the future, we may propose postoperative staged treatment options for patients with postoperative urinary incontinence after radical prostate cancer surgery. Researchers have also found acupuncture to be more effective for pelvic floor muscle treatment (75), pending further development of the database in the future experimental results in different languages can be cross-referenced, and future researchers may add to this paper for acupuncture treatment, future researchers may add a comparison of the effects of acupuncture treatment to this paper.

The majority of randomized controlled trials that are currently available in clinical practice have been entered in this study, but there may still be some omissions or errors in the analysis. Most of the clinical data in this paper are usually published in professional journals and magazines. For conference papers, due to the difficulty of finding the original text, the data extracted from other journals may be inaccurate, and only some of the conference papers where the original text can be found are used in this paper. As some of the experiments may have different conditions, there may be some contradictions between experiments, we have reduced the influence of potential influencing factors on the analysis results after a more formal and reasonable method.

In this study, we mainly discussed the effect evaluation of various treatment methods for urinary incontinence after radical prostate cancer surgery. The baseline level of urinary incontinence patients is also a confounding factor affecting postoperative urinary incontinence, which mainly affects the evaluation of treatment effect alone, whereas the baseline level of urinary incontinence has little impact on the comparison of multiple treatment methods. There is still a large scope for research in this area of clinical research, and there are gaps in the study of many treatment modalities that need to be investigated in more depth.

## 5 Conclusion

In this network meta-analysis, we compared the efficacy of pelvic floor muscle training-based pelvic floor therapy measures in patients with postoperative urinary incontinence after radical prostate cancer surgery. We observed that biofeedback + professional therapist–guided treatment demonstrated superior therapeutic efficacy at 1 month to 6 months for early recovery of incontinence, and electrical nerve stimulation therapy demonstrated superior efficacy at about 3 months postoperatively for recovery of incontinence in the middle of the postoperative period. In December postoperatively, no significant difference was observed in the rest of the modalities, except for electrotherapy + biofeedback + professional guidance. Thus, we can conclude that electrostimulation and biofeedback have a better effect in the early and middle postoperative period, and if they are not effective, pelvic floor muscle training + routine care + biofeedback + professional therapist–guided treatment + electrical nerve stimulation therapy is still an effective measure to recover incontinence in the longer postoperative period of about 1 year. In 12 months, the various modalities do not show much variation. In the cost-effectiveness of treatment, pelvic floor muscle training + routine care + biofeedback + professional therapist–guided treatment + electrical nerve stimulation therapy within 3 months can quickly restore urine control, improve patients’ quality of life and the cost of follow-up daily care. After 3 months, pelvic floor muscle + routine care may be a more economical option to treat urinary incontinence.

It can greatly reduce the cost of treatment. However, with this type of treatment, patients may experience a decrease in quality of life and may increase the cost of care during urine-controlled recovery time.

## Data availability statement

The original contributions presented in the study are included in the article/**Supplementary Material**. Further inquiries can be directed to the corresponding author.

## Author contributions

KY: Writing – original draft. FB: Data curation, Formal analysis, Writing – original draft. TJ: Investigation, Methodology, Writing – review & editing. ZL: Supervision, Validation, Writing – review & editing. RH: Data curation, Formal analysis, Supervision, Writing – original draft. SC: Writing – review & editing, Methodology, Supervision. JL: Conceptualization, Formal analysis, Writing – review & editing.
