# Supplementary figures and images for: Urinary incontinence rehabilitation of after radical prostatectomy: a systematic review and network meta-analysis
Source: Front Oncol. 2024 Mar 22;13:1307434. doi: 10.3389/fonc.2023.1307434 (PMC10996052; doi:10.3389/fonc.2023.1307434)

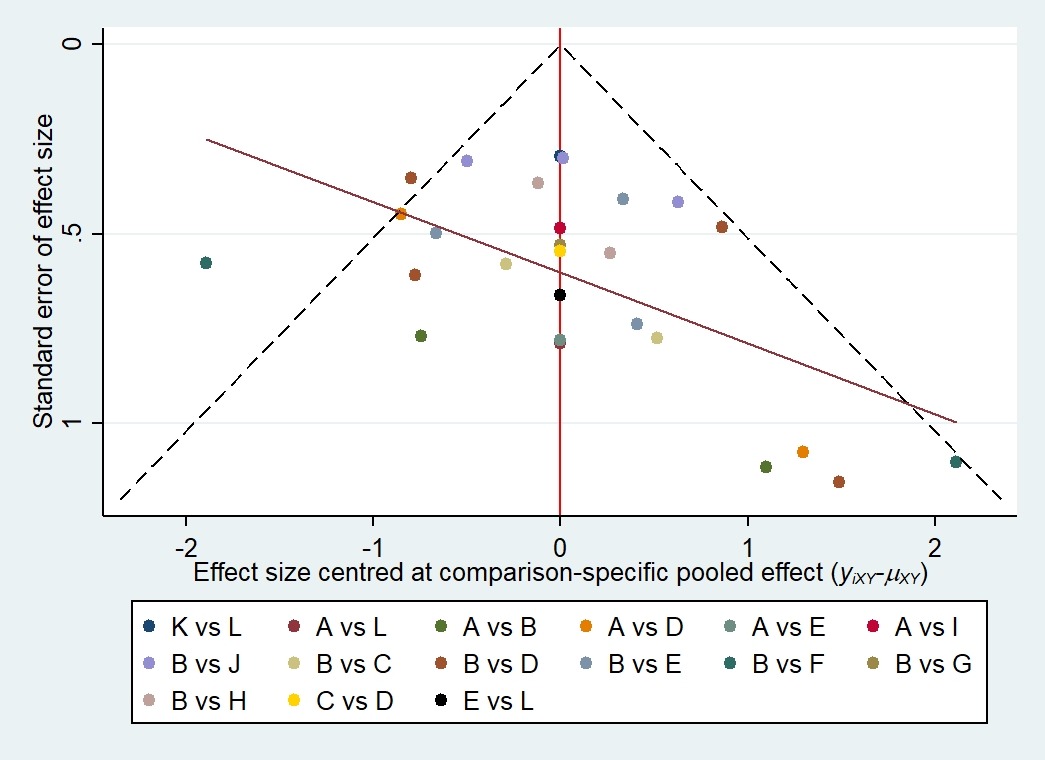

Supplement: Supplementary file 2 [file Image_1.tif]

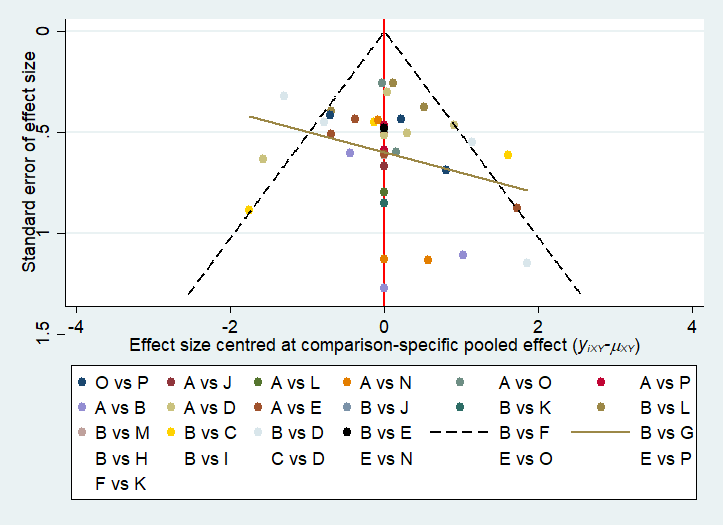

Supplement: Supplementary file 3 [file Image_2.tif]

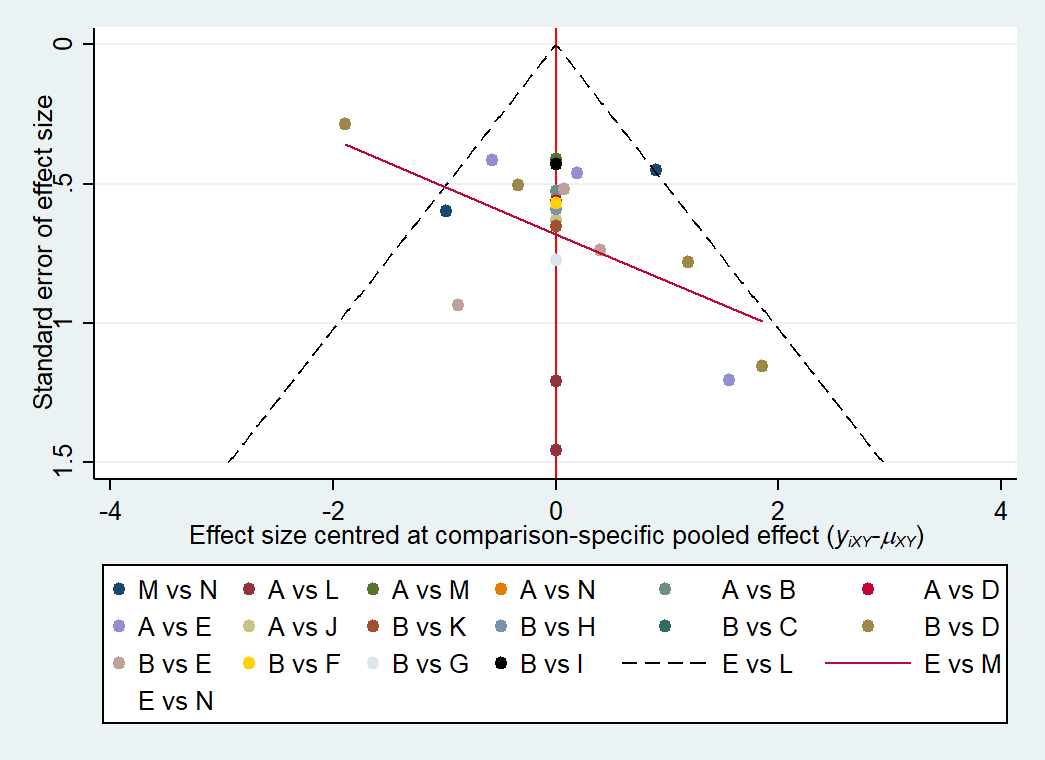

Supplement: Supplementary file 4 [file Image_3.tif]

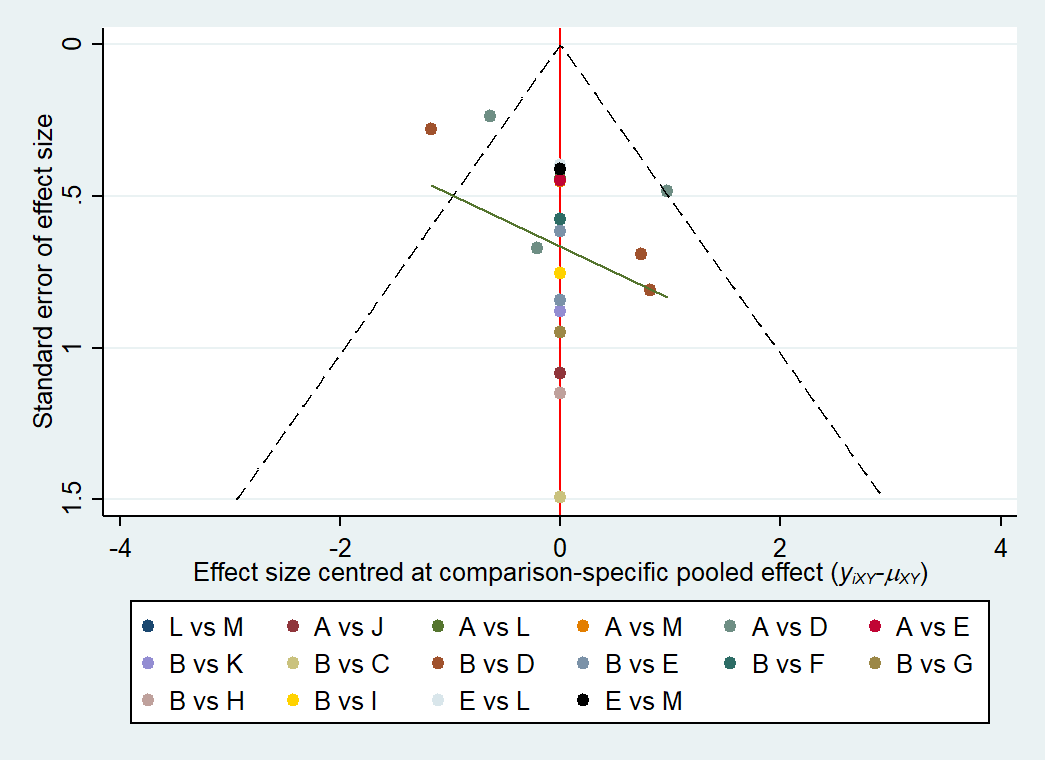

Supplement: Supplementary file 5 [file Image_4.tif]
